# Training and illness characteristics of cross-country skiers transitioning from junior to senior level

**DOI:** 10.1371/journal.pone.0250088

**Published:** 2021-05-14

**Authors:** Øyvind Karlsson, Marko S. Laaksonen, Kerry McGawley

**Affiliations:** Department of Health Sciences, Swedish Winter Sports Research Centre, Mid Sweden University, Östersund, Sweden; Universita degli Studi di Verona, ITALY

## Abstract

**Objective:**

This study aimed to describe the endurance training and incidence of illnesses reported by a group of well-trained cross-country (XC) skiers throughout their transition from junior to senior level.

**Methods:**

Changes in self-reported training and performance, from 31 well-trained XC skiers, were analyzed from the start of the season they turned 16 y until the end of the season they turned 22 y, using linear mixed-effects models. Differences in the incidence of self-reported illness episodes were analyzed using incidence rate ratios, and the relationships between self-reported illness and training volumes were analyzed using linear mixed-effects models in a sub-group of 23 of the skiers.

**Results:**

In total, 145 seasons of training data (including 85,846 h of endurance training) and 109 person-years of illness data (including 380 self-reported illness episodes) were analyzed. The athletes progressively increased their annual endurance training volume from age 16 to 22 y in a linear fashion, from ~ 470 to 730 h. Low- and high-intensity training volumes increased by 51.4 ± 2.4 h·y^-1^ (p < .001) and 4.9 ± 0.6 h·y^-1^ (p < .001), respectively. Sport-specific and non-specific training increased by 50.0 ± 2.2 h·y^-1^ (p < .001) and 4.6 ± 2.0 h·y^-1^ (p < .001), respectively. The athletes reported a median (range) of 3 (0–8) illness episodes and 17 (0–80) days of illness per year, and there was an inverse relationship between self-reported illness days and annual training volume (-0.046 ± 0.013 d·h^-1^; p < .001).

**Conclusions:**

This group of well-trained XC skiers increased their endurance training volume in a linear fashion by ~ 55 h annually. This was primarily achieved through an increase in low-intensity and sport-specific training. Furthermore, higher training volumes were associated with a lower number of self-reported illness days.

## Introduction

Competing at a world-class level in any endurance sport requires a large volume of systematic training performed over time. Elite senior cross-country (XC) skiers typically perform 650–950 h of annual training distributed across 400–500 sessions, where ~ 85–95% of the training is endurance based [1–4]. Total training volume appears to be highest during the general preparation phase (June–August), with a subsequent decrease in training volume during the specific preparation (September–November) and competitive (December–March) phases [1, 2]. Consistent with training-volume distribution, training frequency is at its highest during the general preparation phase, with a gradual reduction towards and throughout the competitive season [1, 2]. In contrast, the volume [1] and frequency [2] of high-intensity training, specifically, appear to increase throughout the season. In general, elite senior XC skiers typically apply a “polarized” training-intensity distribution, with the majority (~ 80–90%) of their endurance training performed below the first lactate threshold (LT) [2, 4, 5]. Regarding exercise mode, the proportion of ski-specific exercise increases considerably from the general preparation phase (~ 50–60%) to the competition phase (~ 80%) [1, 2].

While the training characteristics of senior elite-level XC skiers are well-documented in the literature [1, 2, 6, 7], limited up-to-date information exists on the long-term training characteristics of XC skiers transitioning from junior to senior level. Except for a recent case study describing the long-term training practices of the world’s most successful female XC skier [8], the only documentation of long-term training practices (i.e., monitoring over > 1 season) was published almost 30 years ago [9]. That study reported the training practices of 41 Finnish XC skiers between the ages of 14–24 y and showed that total weekly training distance increased from 40 km·week^-1^ at age 14 y to 140 km·week^-1^ at age 24 y. Skiing, roller-skiing, and running comprised ~ 40%, 45%, and 15% of the total distance, respectively, with no significant differences in these ratios between ages. When considering the extensive changes that have occurred in XC skiing over the last 30 years [4], particularly with respect to the rules, competitive events on offer, and technological and technical advancements, a scientific update is overdue. Moreover, since the transition from junior to senior level is a critical phase in an athlete’s development, this period warrants further investigation.

In order to attain the high training volumes required to compete at a world-class level in endurance sports, resilience against acute illness is important [10, 11]. However, it has been suggested that the relationship between training volumes and illness (particularly upper-respiratory-tract infections, UTRIs) follows a “J-curve” [12], where the risk of illness decreases with moderate training volumes and increases again with high training volumes, as are typical of elite endurance athletes. The “Open Window” hypothesis [13–15] has been proposed to explain this phenomenon, with an acute suppression of the immune system following intense and prolonged exercise (for a review, see [16]), placing the athlete at greater risk of infection during this “window of opportunity”. While both the “J-curve” and “Open Window” hypotheses have been popular, evidence-based frameworks within the exercise immunology disciplines for decades [17, 18], more recent research challenges the notion that intense and prolonged exercise affects immune function and increases susceptibility to illness in this way [19, 20]. Nevertheless, maintaining immune health is a challenge for elite athletes who are subjected to extreme physical and mental demands, and other potential stressors such as frequent air travel, congested competition schedules, low energy availability, sleep disturbances and viral epidemics. For XC skiers in particular the constant exposure to cold air and lack of sunlight may be further threats to maintaining good health. On average, elite senior XC skiers report 3–4 illness episodes per year, with an average duration of approximately 4 days per episode [21], which is similar to that reported in elite swimmers [22]. The time of season seems to influence the duration and frequency of illness episodes, with these athletes reporting the highest frequency of symptoms during the winter months [21, 22]. While there are considerable inter-individual variations in the number of reported illness events and days per year, higher-performing skiers have been documented to experience significantly fewer illness days [21]. However, the illness patterns in young XC skiers have not to date been documented or compared to those of their senior counterparts.

The primary aim of the current study was to describe the endurance training undertaken by a cohort of well-trained XC skiers transitioning from junior to senior level. The secondary aim was to describe the incidence of self-reported illness and the relationships between training volume and self-reported illness in this cohort.

## Materials and methods

### Participants

A group of 31 well-trained XC skiers (12 females and 19 males) was recruited via convenience sampling using the following criteria: (1) selected by the Swedish Ski Association to represent the national senior, development or junior team in XC skiing, or one of the specialist ski universities in Sweden, at the time of inclusion; (2) over 18 years of age at the time of inclusion; (3) having recorded day-to-day training in detail for at least 2 complete seasons between the end of the 2011/2012 and 2017/2018 training and competitive seasons. In total, 26 of the skiers (10 females and 16 males) had been selected to represent at least one of the Swedish national teams and 7 of the skiers (5 females and 2 males) had won at least one individual U23 or Junior World Championship medal. Of the 31 skiers recruited to the study, training-diary information relating to illness episodes was available for analysis in a sub-group of 23 skiers (11 females and 12 males). The remaining 8 skiers were excluded from this sub-group due to a failure to report illness information consistently. All participants were fully informed about the nature of the study before providing written consent for their data to be included. The study was pre-approved by the regional ethical review board in Umeå, Sweden (reference: 2018-46-31M).

### Study design

**Fig 1:**
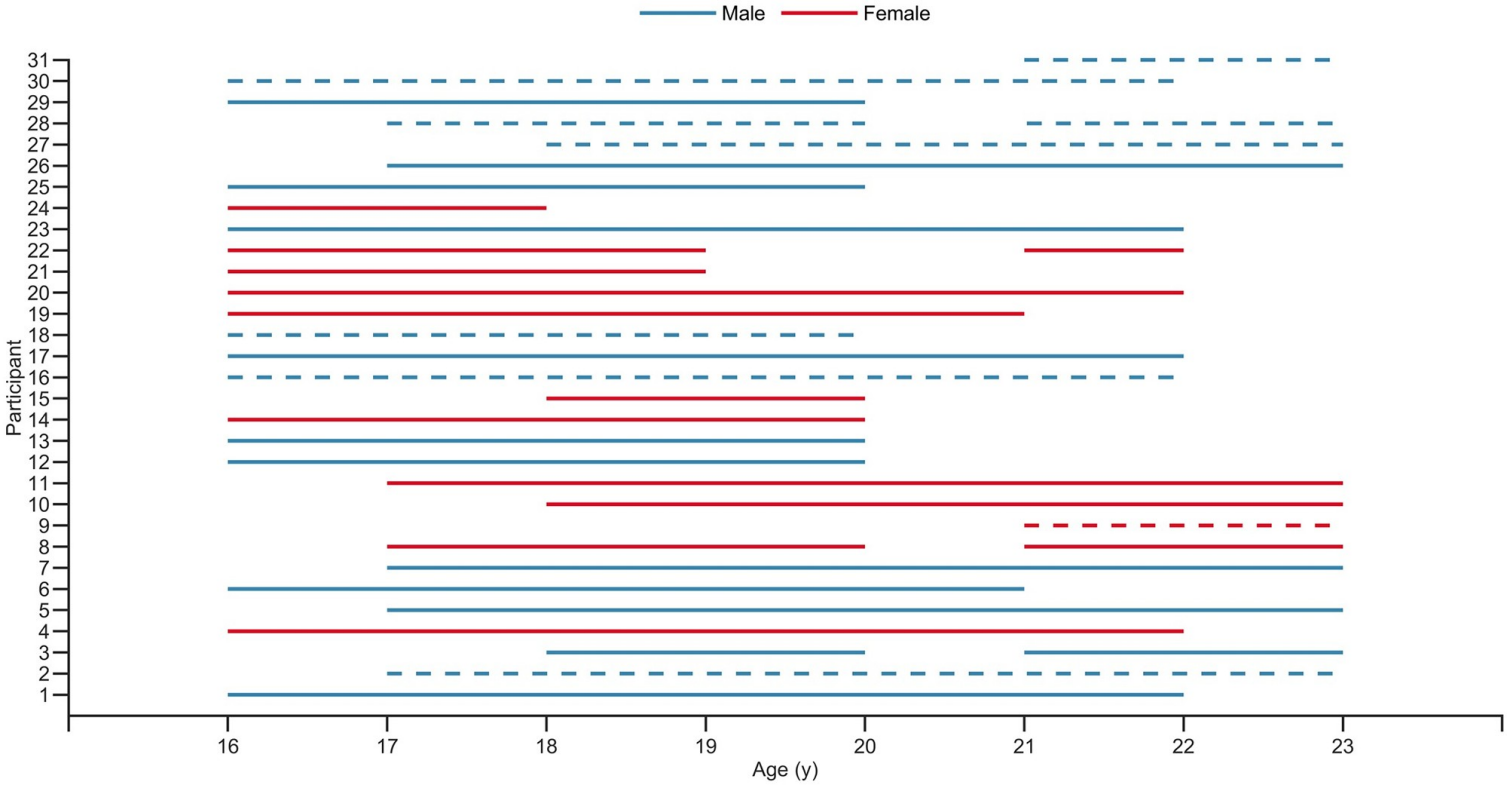
Annual data included in the final analysis for each participant. Solid lines: training and illness data; dashed lines: training data only.

A retrospective cohort study design was used to collate self-reported training and illness data recorded between 23^rd^ April 2012 and 22^nd^ April 2018. Individual training and illness data were retrieved for each skier from the season starting the year they turned 16 y until the season starting the year they turned 22 y. Explorative data analyses were subsequently performed. The annual data included for each skier is illustrated in Fig 1.

### Training data

The skiers recorded their day-to-day training in a bespoke online training diary developed specifically for the Swedish Ski Association. An endurance training session was defined as a session containing at least 60 minutes of exercise, including warm-up and cool down, and training intensities were categorized according to the 4-zone intensity scale developed by the Swedish Ski Association [23], as presented in Table 1 (values in this table are guidelines only and athletes made their own subjective adjustments during training). The 60-minute threshold was chosen after thorough inspection of the data and extensive discussions with the athletes and their coaches regarding training session goals and formats. The information recorded for endurance training included total training times in different activities (on-snow XC skiing, roller skiing, running, cycling, orienteering, ski-walking, or “other”) and intensities (according to the 4 zones defined in Table 1). On-snow skiing and roller skiing were classified as sport-specific training while running, cycling, orienteering, ski-walking and “other” were defined as non-specific training.

**Table 1:** The 4-zone intensity scale used by the Swedish Ski Association and the binary intensity scale used for categorization of endurance training in the current study.

Intensity zone	Reference heart rate (% maximum)	Binary scale	Examples of training
4	> 95	HIT	Competitions, high-intensity interval training with recovery time equal to 50–100% of work interval time. Total training time in this zone: 20–30 min.
3	85–95	HIT	Natural intervals, longer intervals with recovery time equal to 25–50% of work interval time. Total training time in this zone: 30–60 min.
2	75–84	LIT	Moderate-intensity continuous training, natural intervals. Total training time in this zone: 45–90 min
1	60–74	LIT	High-volume low-intensity continuous training, recovery session and warm-up. Effective training time < 180 min.

Abbreviations: HIT: high-intensity training; LIT: low-intensity training.

NB. The text has been translated from Swedish for the purposes of this communication.

The skiers allocated training time to each intensity zone using a modified session goal approach [24] based on the primary goal of the session and recordings from their personal heart rate (HR) monitors. Since the 4 intensity zones are not defined by underlying physiological events [25], the binary model presented by Tønnesen et al. [2] was adopted before analysis of the data. In this model, low-intensity training (LIT) refers to training intensities < 85% of the maximal HR (to approximate sub-LT training) and high-intensity training (HIT) refers to training intensities ≥ 85% of the maximal HR (to approximate supra-LT training). The information recorded for strength training included total training time and type (general, specific or maximal), while for speed training the number and type (short, long or incremental) of sprints performed was recorded. Total time was also recorded for mobility/flexibility training.

Consistent with the practice of the Swedish Ski Association, each annual cycle was divided into 13 x 4-week training periods, starting from week 17 of the respective calendar year (i.e., late April). The 13 training periods were grouped into 5 periodization phases (transition, general preparation, specific preparation, competition and regeneration) each lasting different durations (Table 2).

**Table 2:** A description of the 5 periodization phases across the annual cycle.

Periodization phase	Training period	Time of year	Number of days
Transition	1	Late April–Late May	28
General preparation	2–7	Late May–Early November	168
Specific preparation	8–9	Early November–End December	56
Competition	10–12	Start January–Late March	84
Regeneration	13	Late March–Late April	28

### Illness data

An illness episode was defined as any self-reported event of illness incurred, at any point in time, regardless of the consequences with respect to absence from training or competition, as based on the definition from the International Olympic Committee consensus statement [26]. Athletes reported illness episodes in their online training diaries (injuries were reported separately) and in order to be categorized as separate events, illness episodes had to be separated by a minimum of 7 days [21, 22].

### Performance level

Performance level was determined using the FIS (International Ski Federation) points system, whereby a skier’s FIS points score at any given time is calculated as the average top 5 results (in FIS points) over the last 365 days (an adjustment factor of > 1.0 is applied if fewer than 5 results are available) [27]. The number of FIS points gained in a single competition is determined by adding the individual race points (P) to the race penalty score, where P is calculated as follows:

$$P=\frac{F\times T_{x}}{T_{o}}-F$$ (1)

F is the race factor (800 for all individual time trials; 1200 for sprints and pursuit races; 1400 for mass start and skiathlon races), *T*_*x*_ is the race time (in seconds) and *T*_*o*_ is the winning race time (in seconds). Hence, lower race points indicate a better performance. The race penalty is calculated by summing the 3 highest FIS points scores (from the current FIS points list) from the top 5 finishers in the respective competition, then dividing by 3.75. Separate FIS points lists are used for distance and sprint competitions, and these are publicly available at the FIS website [28].

### Statistical analyses

Training data are presented as mean ± standard error (SE), unless otherwise stated, while illness data are presented as median (range). All statistical tests were carried out with the software R (R Core Team, 2020) and the alpha level was set at p < .05 for all tests. Linear mixed-effects models were fitted by restricted maximum likelihood, to assess the relationships between total annual training volume, LIT volume, HIT volume, number of endurance training sessions, specific and non-specific training volume, performance level and age. Separate models were constructed for each dependent variable with age as a fixed effect and skier as a random effect, with both random intercepts and slopes to account for repeated measures. Linear mixed-effects models were also fitted to assess the effect of total training volume on performance while controlling for age. Separate models were constructed for distance and sprint performance, with total training volume and age as fixed effects and skier as a random effect, with both random intercepts and slopes for the sprint model and random intercept only for the distance model. Finally, a linear mixed-effects model was fitted to assess the relationship between annual number of self-reported illness days and LIT volume, HIT volume and age. LIT volume, HIT volume and age were fitted as fixed effects, while skier was fitted as a random effect, with random intercepts to account for repeated measures. Sex was not included as a fixed effect in any of the models due to the overall low number of participants.

Model selection relied on Akaike’s information criterion (AIC). Model assumptions were checked by visual inspection of residual plots. Influential data points were assessed using standardized DFBETAS (> 1) and Cook’s distance (> 1) with the influence.ME package [29]. The lme4 package [30] was used to fit all linear mixed-effect models. P values were derived using Satterthwaite approximations with the lmerTest package [31], as recommended by Luke [32], while 95% confidence intervals (CIs) were obtained using the profile likelihood method. Conditional and marginal R^2^ values for the linear mixed-effects models were calculated according to Nakagawa et al. [33].

Differences in the incidence of self-reported illness episodes were analyzed using incidence rate ratios calculated with the epitools [34] package. To compare the incidence of self-reported illness at different ages all cases amongst the junior skiers (age < 19 y) were compared with all cases amongst the senior skiers (age ≥ 19 y), due to a limited number of participants in some age groups preventing year-by-year analyses. To compare the incidence of self-reported illness episodes between spring (March–May), summer (June–August), autumn (September–November) and winter (December–February), as well as between the 5 periodization phases (see Table 2), illness data were pooled across the entire 7-y period before analysis. Differences in the total number of illness days per year and average duration of illness episodes between the two age groups were analyzed using Wilcoxon rank-sum tests. The association between total number of self-reported illness days and total training volume was determined using repeated-measures correlation (*r*_*rm*_), which were carried out using the rmrcorr package [35].

## Results

In total, 145 seasons of training data were analyzed for the 31 skiers, containing 44,906 individual endurance training sessions (representing 82% of the total number of training sessions registered) and 85,846 h of endurance training. For the sub-group of 23 skiers for which illness data were reported, 109 person-years of data revealed 380 self-reported illness episodes and 2,129 days of self-reported illness. Prior to analysis, two complete seasons were excluded for one athlete owing to a confirmed eating disorder, while data for previous and/or subsequent seasons were retained for this individual.

### Annual training characteristics

**Fig 2:**
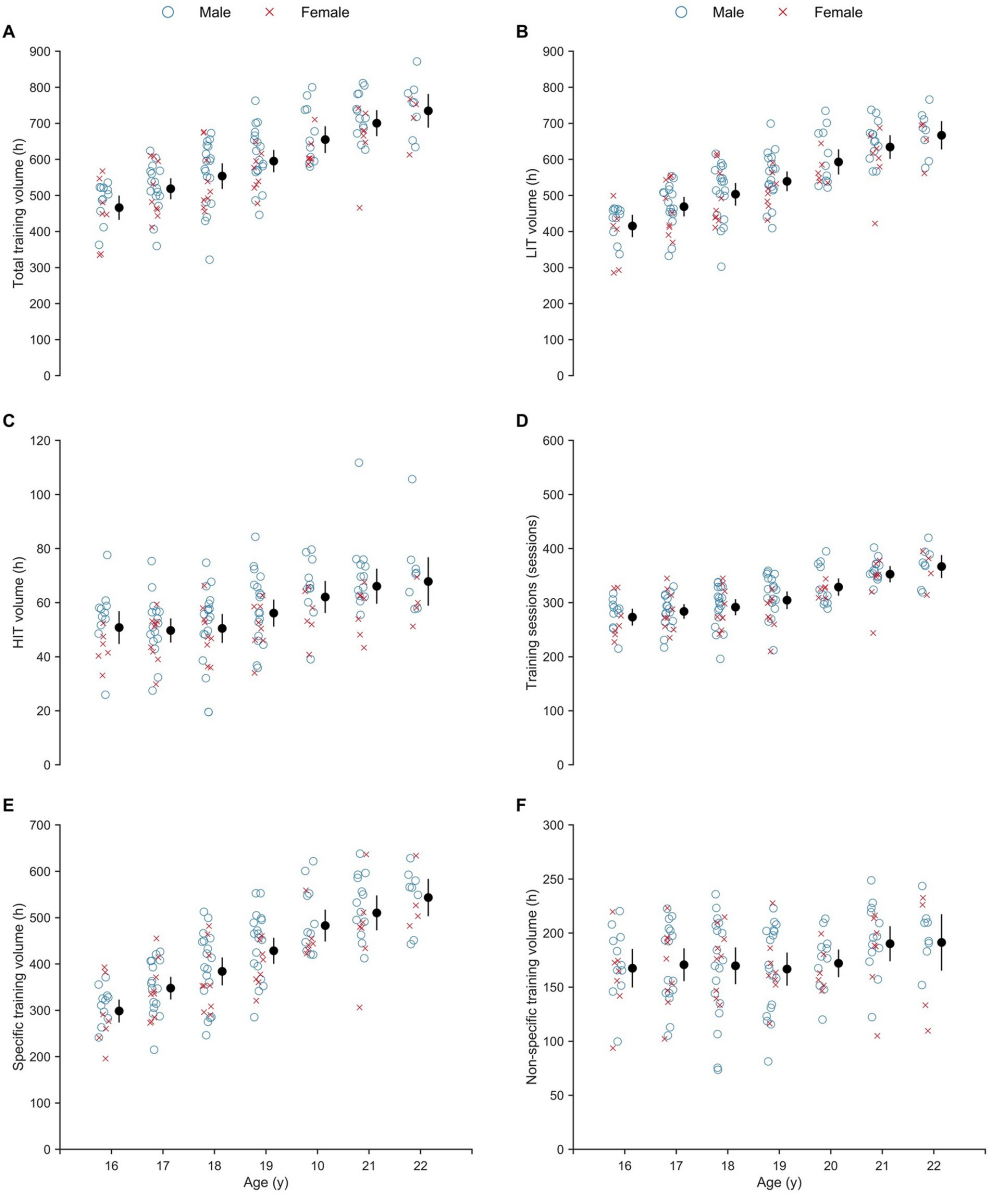
Progression in annual endurance training from age 16 to 22 years (y) old. (A) total annual training volume; (B) low-intensity training (LIT) volume; (C) high-intensity training (HIT) volume; (D) number of endurance training sessions; (E) specific (i.e., on-snow skiing and roller skiing) training volume; and (F) non-specific (i.e., running, cycling, orienteering, ski-walking and “other”) training volume. Closed circles and whiskers: mean ± 95% CI.

Total annual endurance training volume, LIT volume, HIT volume, number of endurance training sessions (including competitions) and specific/non-specific training sessions from age 16 to 22 y are presented in Fig 2. Over this transition period, total annual endurance training volume increased by ~ 75% (55.9 ± 2.8 h·y^-1^; 95% CI [50.1, 61.6]; t (23.380) = 19.84; p < .001), with total annual LIT and HIT volume increasing by ~ 75% and ~ 60%, respectively (51.4 ± 2.4 h·y^-1^; 95% CI [46.4, 56.1]; t(22.520) = 21.35; p < .001 and 4.9 ± 0.6 h·y^-1^; 95% CI [3.8, 5.9]; t(26.832) = 8.96; p < .001, respectively). The number of endurance training sessions increased by ~ 50% (21 ± 1 sessions·y^-1^; 95% CI [18, 24]; t(22.332) = 15.02; p < .001) and the amount of specific and non-specific training increased by ~ 110% and ~ 20%, respectively (50.0 ± 2.2 h·y^-1^; 95% CI [45.4, 54.4]; t(25.508) = 22.74; p < .001 and 4.6 ± 2.0 h·y^-1^; 95% CI [0.5, 8.5]; t(27.435) = 2.89; p < .030, respectively).

### Performance level

**Fig 3:**
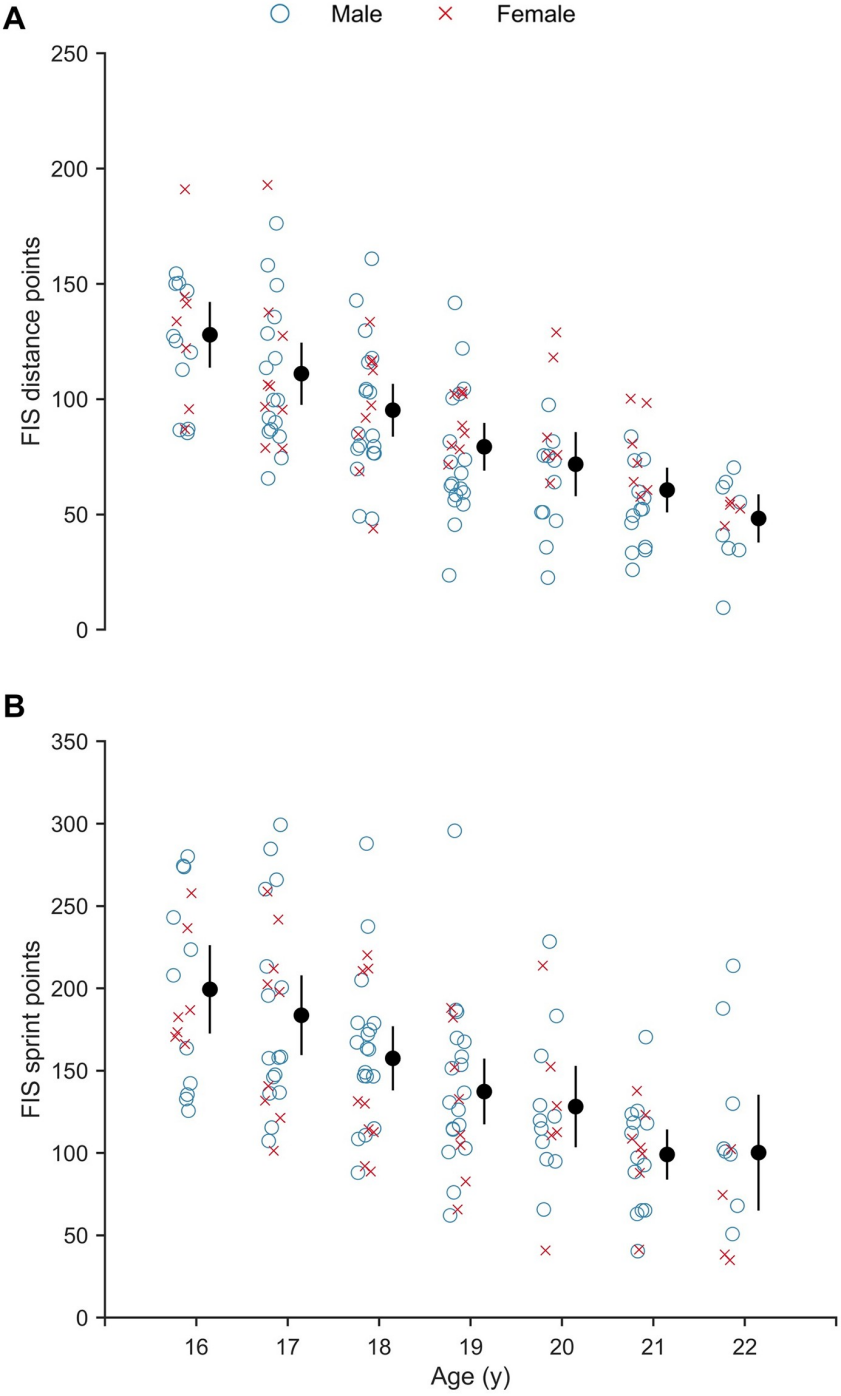
Progression in performance level from age 16 to 22 years (y) old represented by FIS points. (A) distance and (B) sprint competitions. Closed circles and whiskers: mean ± 95% CI.

The progression in distance and sprint FIS points from age 16 to 22 y is presented in Fig 3, which shows distance points to decrease by ~ 65% (14.2 ± 0.8 points·y^-1^; 95% CI [-15.8, -12.6]; t(124.297) = -17.31; p < .001) and sprint points to decrease by ~ 55% (19.0 ± 2.1 points·y^-1^; 95% CI [-23.4, -14.9]; t(22.232) = -9.09; p < .001).

Modeling the relationships between total training volume and FIS points, while controlling for the effect of age, showed that both total training volume and age had a statistically significant positive effect on distance performance (i.e., a reduction in FIS points), while only age had a statistically significant effect on sprint performance (Table 3).

**Table 3:** Fixed-effects estimates (B) with 95% confidence intervals (CI), standard errors (SE) and p values for the effects of total training volume and age on performance level represented by FIS points for distance and sprint competitions.

	B	95% CI	SE B	p	cR^2^	mR^2^
**FIS distance points**					.82	.49
Intercept	162.25	135.68, 188.86	13.62	< .001		
Age	-9.68	-13.68, -6.17	1.79	< .001		
Total training volume	-0.08	-0.14, -0.03	0.03	< .001		
**FIS sprint points**					.82	.33
Intercept	208.75	153.69, 261.62	26.53	< .001		
Age	-17.67	-25.02, -10.34	3.70	< .001		
Total training volume	-0.03	-0.14, 0.09	0.06	.643		

Note. cR^2^ = conditional R^2^; mR^2^ = marginal R^2^.

### Self-reported illness

On average, the skiers reported 3 (0–8) episodes and 17 (0–80) days of illness per year over the analyzed time period. The average duration of illness episodes was 4 (1–60) days, with the most commonly reported reason being symptoms of a URTI. The incidence of self-reported illness episodes was higher at age 16–19 y compared with 19–22 y (3.9 vs. 3.1 episodes per person-year, IRR = 0.80, 95% CI [0.66, 0.98], p = .033). However, the average duration of illness episodes (4 [1–24] vs. 4 [1–60] days, *U* = 40688, z = -0.4346, p = .663) and total number of illness days (20 [0–47] vs. 17 [0–80], *U* = 2722, z = -1.1680, p = .243) did not differ between the junior and senior skiers.

**Fig 4:**
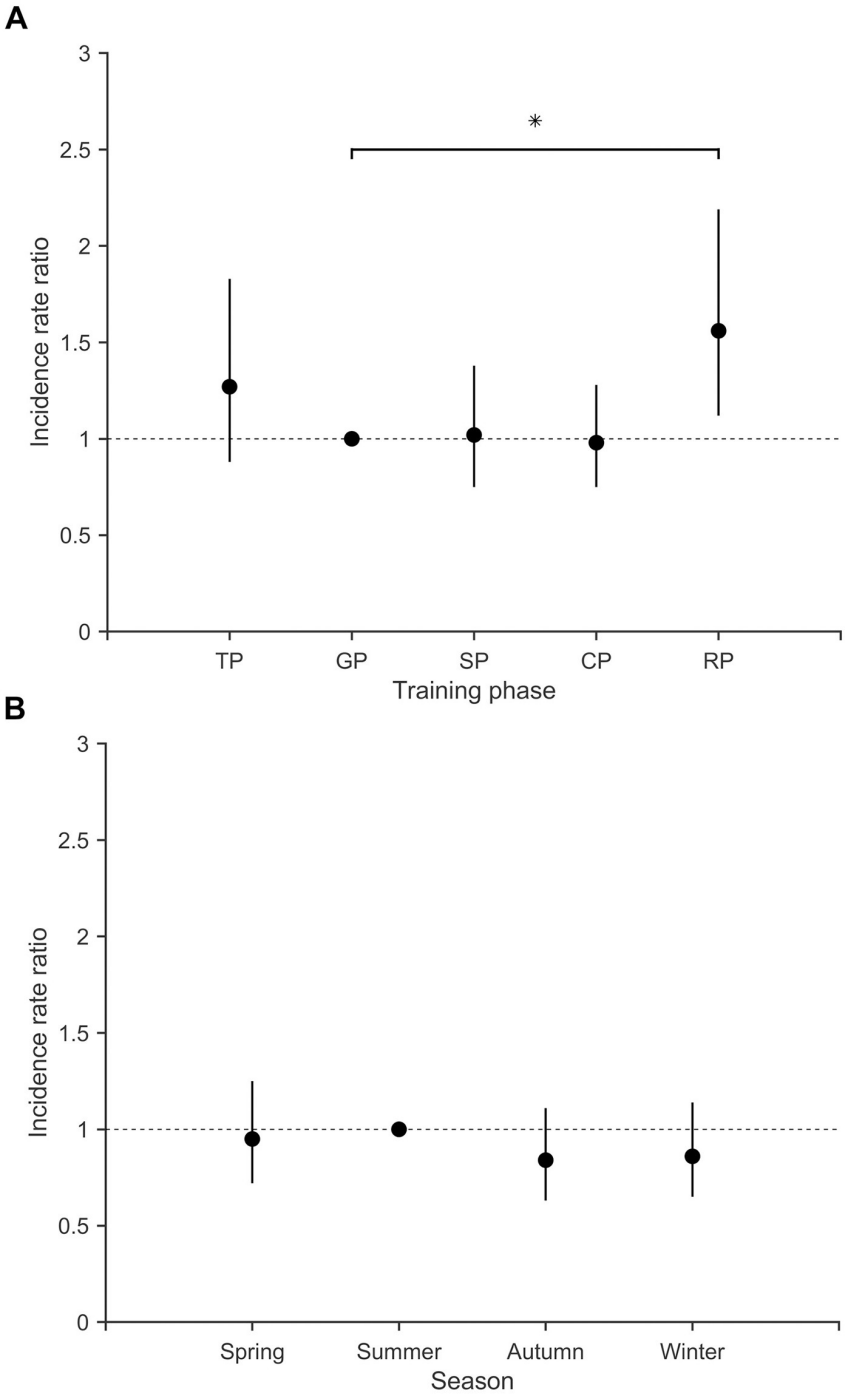
Incidence rate ratios with 95% CIs comparing incidence across training phases and seasons. (A) training phases, comparing the specific preparation (SP), competition (CP), regeneration (RP) and transition phase (TP) to the general preparation phase (GP, referent category), and (B) seasons, comparing the spring, autumn and winter to the summer (referent category). * significantly different from the respective referent category (p < .05).

Incidence rate ratios calculated to compare rates of self-reported illness across training phases and seasons using the general preparation phase and summer as referent categories are displayed in Fig 4. The regeneration phase was associated with a higher rate of self-reported illness episodes compared to the general preparation phase (1.4 vs. 0.9 episodes per 100 person-days, IRR = 1.56, 95% CI [1.12, 2.19], p = .009), while there were no differences in incidence between the other training phases or seasons (all p >.200).

**Fig 5:**
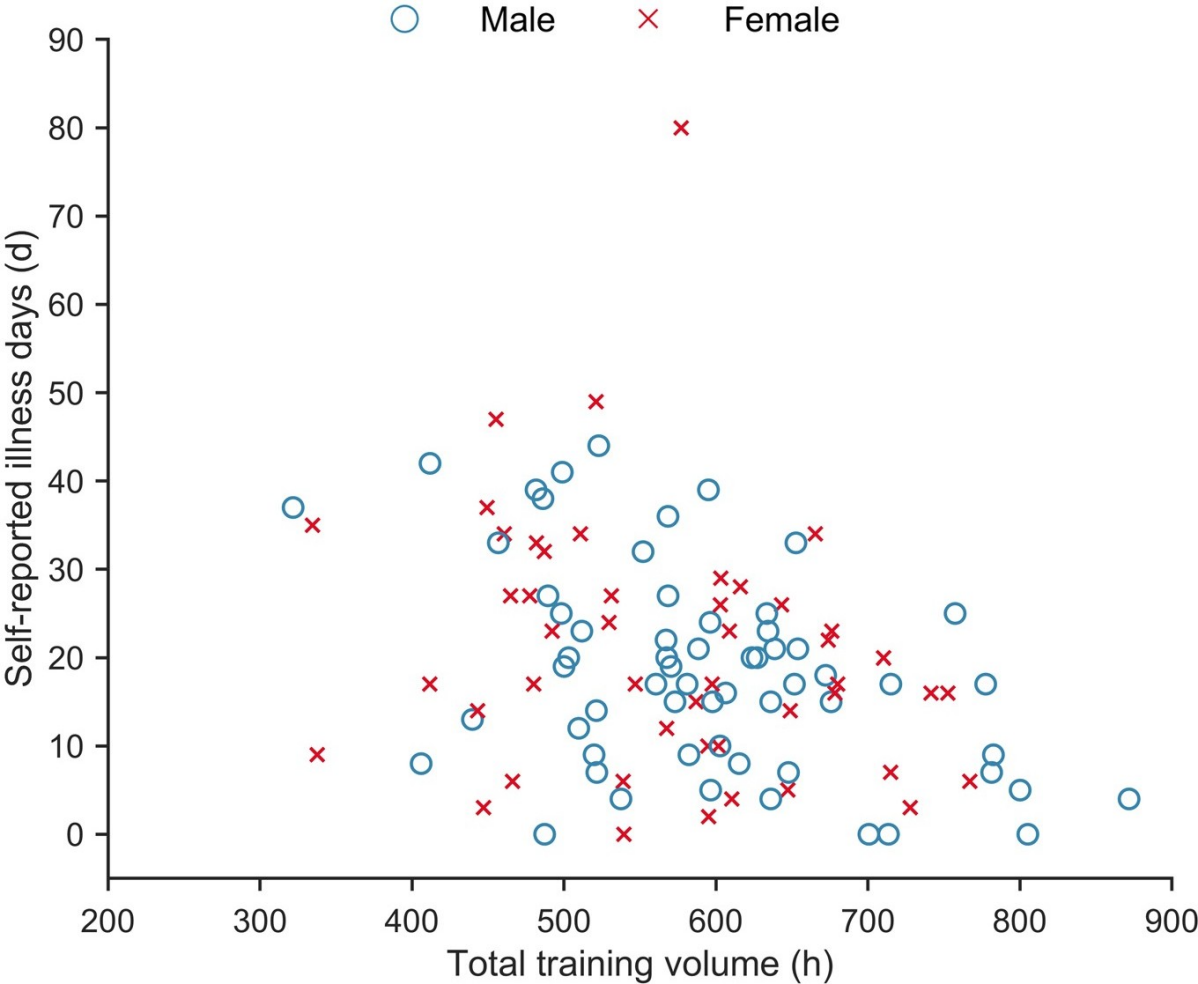
Association between self-reported illness days and total annual training volume.

The relationships between the annual number of self-reported illness days and LIT volume, HIT volume and age are presented in Table 4. Both LIT and HIT volume had a statistically significant negative relationship with the number of self-reported illness days, while age had a statistically significant positive relationship with the number of self-reported illness days. The association between the annual number of self-reported illness days and total annual training volume (*r*_*rm*_ = -.34; 95% CI [-.51, -.13]; p = .002) is displayed in Fig 5.

**Table 4:** Fixed-effects estimates (B) with 95% confidence intervals (CI), standard errors (SE) and p values for the effects of low-intensity training (LIT) volume, high-intensity training (HIT) volume and age on number of self-reported illness days.

	B	95% CI	SE B	p	cR^2^	mR^2^
**Illness days**					.41	.21
Intercept	55.27	39.49, 71.11	8.13	< .001		
LIT	-0.05	-0.10, -0.03	0,02	.001		
HIT	-0.14	-0.26, -0.02	0,06	.023		
Age	2.29	0.27, 4.31	1,04	.031		

Note. cR^2^ = conditional R^2^; mR^2^ = marginal R^2^.

## Discussion

The purpose of this study was to examine the long-term endurance training and illness characteristics in a group of well-trained XC skiers transitioning from junior to senior level. The main findings showed that, over the 7-y observation period: (1) the skiers progressively increased their annual endurance training volume in a linear fashion from ~ 470 h to ~ 730 h; (2) this increase in total endurance training volume was primarily achieved through an increase in LIT and sport-specific training, rather than HIT and non-specific training; (3) both training volume and age significantly influenced performance level, which also improved in a linear fashion; (4) higher training volumes were associated with a lower number of self-reported illness days.

### Annual training characteristics

The accumulation of high training volumes is a common factor among top senior, elite-level XC skiers [2, 3, 8, 36]. From the age of 16 to 22 y, the athletes in the present study progressively increased their annual endurance training volume by ~ 56 h·y^-1^, to ~ 730 h at age 22 y. This latter figure is comparable to previous reports of endurance training volumes in senior, elite-level XC skiers [1–3, 6, 36], and has also been reported as “sufficient” to achieve international success at Olympic, World Championship and World Cup levels [2, 8]. However, at 22 y the mixed-sex cohort of skiers in the present study still performed ~ 100 h less endurance training than the training volumes previously reported for truly world-class female XC skiers who have consistently performed at the very highest international level [6, 8]. Specifically, it was not until the world’s most successful female XC skier reached annual endurance training volumes in excess of 800 h that she performed consistently at a world-class level in both distance and sprint races, despite having previously performed at a world-class level in sprint skiing with annual training volumes of ~ 700 h [8]. Hence, the data suggest that the skiers observed in the current study appeared to reach a volume of endurance training within the analyzed period that would be sufficient to perform at an elite senior level in XC skiing, but that a further increase in endurance training volume may be necessary to produce stable and consistent results that enable success at a top world-class level.

The skiers in the present study progressively increased their endurance training volume in a linear fashion throughout the 7-y observation period. By contrast, Solli et al. [8] has previously reported that the world’s most successful female XC skier increased her training volume in a non-linear fashion from age 20 y until her most successful 5-year period. Similarly, Pinot and Grappe [37] reported that a top-10 cycling Grand Tour finisher increased his training volume in a non-linear fashion, with a 60% increase from age 18 to 20 y followed by a 10% increase from age 20 to 23 y. However, data presented by Rusko [9] indicates that a group of Finnish XC skiers, who all later represented the Finnish national team in XC skiing, increased their weekly training distance (including running, skiing, roller skiing and ski-walking) in a linear fashion from age 16 to 24 y. While both the current and previous data support that a gradual increase in training volume is beneficial, further research is needed to determine the optimal way of organizing the rate of this progression in developing athletes.

While the absolute volume of LIT increased by more hours per year than HIT throughout the observed period, the proportions of LIT and HIT remained stable at around 90:10. By age 22 y the skiers in the current study were performing ~ 670 h of LIT, which is comparable to world-class female and male XC skiers who have been reported to perform ~ 650 h of LIT [2, 8]. Moreover, two previous studies have reported that world-class male and female skiers performed ~ 30% more LIT than their national-level counterparts [6, 7]. In addition, the most successful female XC skier performed the highest proportion of LIT (~ 780 h) during her most successful years [8]. It has therefore been suggested that extensive LIT contributes to the superior aerobic capacity and performance of world-class XC skiers [6, 7]. Hence, focusing on gradually developing the tolerance for large volumes of LIT seems important for attaining the top level in XC skiing.

The relative proportion of sport-specific training (i.e., skiing and roller skiing) increased from ~ 65% to ~ 75% of the total annual endurance training volume throughout the 7-y observation period. Previous studies of senior elite-level XC skiers have reported that ~ 65% of the total annual training volume (endurance and sprint) is sport specific [2, 8]. Differences in reporting practices may have contributed to the observed differences between the present and previous studies since the present study only included endurance training in the calculation of specific and non-specific training volumes. However, an increase in the use of roller skis and a greater emphasis placed on upper-body strength and endurance training over the last decades [4] might also explain some of the discrepancies. In addition, the environment in which skiers live may be a relevant factor, with skiing on and off snow potentially more accessible (e.g., in terms of weather, traffic, road surfaces, etc.) to some individuals or training groups. Indeed, the majority of the athletes in the present study had year-round access to a roller-ski track and a ski stadium, good road surfaces with relatively little traffic and snow throughout the winter months, making sport-specific training highly accessible. Despite this apparent advantage, a relatively large volume of non-specific endurance training has been suggested as an important factor facilitating high overall training volumes in elite XC skiers [2, 6, 8], where varying the load on different muscle groups may potentially reduce the risk of injuries [6]. Since injury prevalence was not recorded in the present study, the occurrence of injuries over time in relation to training characteristics warrants further systematic investigation.

### Performance level

Physical and physiological developments in athletes aged 16–24 y have been documented in the literature for XC skiers [9, 38] and other endurance athletes such as cyclists [37] and rowers [39]. This is the first study, however, that appears to have documented the longitudinal changes in sport-specific performance in a group of young, developing XC skiers. The performance level (as assessed by FIS points) in both distance and sprint competitions improved linearly during the 7-y observation period. While the improvement in distance performance was related to an increase in both age (by ~ 10 points·y^-1^) and endurance training volume (by ~ 10 points·100 h^-1^), the improvement in sprint performance was only related to an increase in age (by ~ 18 points·y^-1^). A possible explanation for this discrepancy could be that the current models only incorporate endurance training volume, whereas a more detailed analysis of sprint and strength training may be required for the accurate prediction of sprint performance.

### Self-reported illness

The skiers in the present study reported, on average, 3 (0–8) illness episodes per year across the 7-y observation period. The incidence of self-reported illness episodes was, however, slightly higher at age 16–19 y (~ 4 episodes per year) compared to 19–22 y (~ 3 episodes per year). Only two previous studies have investigated the incidence of illness in athletes across multiple seasons; hence little data exists for comparison. However, the present findings are comparable to previous reports of both senior elite-level XC skiers (i.e., 3–4 episodes of respiratory-tract infections and/or gastrointestinal infections) [21], and elite swimmers (4 episodes of all-cause physician-verified illness episodes per year) [22]. Comparing non-specific illness episodes reported in the current study with respiratory-tract and gastrointestinal infections reported by Svendsen et al. [21], and all-cause illness reported by Hellard et al. [22] seems reasonable, given that respiratory-tract and gastrointestinal infections are the most commonly-reported symptoms in athletes. For example, ~ 96% of illness episodes reported by Hellard et al. [22] were either URTIs or pulmonary infections. The findings are also comparable to those of the general population, who typically experience 2–4 URTIs [40, 41] and 0.3–0.4 acute gastrointestinal infections [42] per year. There were no statistically significant differences in the reported duration of illness episodes (4 [1–24] vs. 4 [1–60] days) or the total number of illness days per year (20 [0–47] vs. 17 [0–80]) for the junior vs. senior skiers in the current study, and these counts are similar to those previously reported for senior elite-level skiers [21]. Hence, it seems reasonable to conclude that the frequency of illness episodes in this group of developing XC skiers is not markedly different from the frequency for either senior elite-level athletes or the general population. Furthermore, episode duration and the total number of illness days per year appear to be comparable between junior- and senior-level XC skiers.

In the present study, higher training volumes were associated with lower numbers of self-reported illness days (Fig 5). These results are in accordance with the results presented by Mårtensson et al. [11], who reported a significant negative correlation between total annual training volumes and self-reported illness days in a mixed group of elite endurance athletes. In contrast, several other studies have reported positive associations between the incidence of URTIs and training load [17, 18], lending support to the notion of a “J-shaped” relationship proposed by Nieman [12]. One possible explanation for this discrepancy might be differences in the applied methods. While both the present study and Mårtensson et al. [11] included all types of illness episodes, Gleeson et al. [17] and Spence et al. [18] only URTIs. Therefore, the current data, consistent with the findings reported by Mårtensson et al. [11], does not support the hypothesis of a “J-shaped” relationship between the incidence of illness and training volume.

In contrast to previous findings in elite endurance athletes, there were no differences in the incidence of self-reported illness between the four seasons. Svendsen et al. [21] and Hellard et al. [22] have previously reported a higher risk of reporting symptoms of acute infections in the northern hemisphere winter months (i.e., September to March) in XC skiers and swimmers, respectively. The current results are also in contrast to the seasonal patterns of URTIs reported in the general population, which have a higher incidence in the winter months [40, 41]. One explanation for this discrepancy might be that the skiers in the present study traveled less by airplane compared to senior elite-level XC skiers, especially during the competition period in the winter months when most of the competitions in their age categories were performed in Sweden during the earlier part and within Scandinavia in the latter part of the 7-y observational period. Another explanation might be that senior elite-level XC skiers have a more condensed competition schedule. Both international air travel and condensed competition periods have previously been associated with a significant increase in the risk of reporting symptoms of illness in XC skiers [21, 43].

### Limitations

The current study design is associated with several limitations that should be taken into consideration when interpreting the data. Firstly, although the study includes large amounts of data from a group of high-performing young athletes over a long period of time, the self-reported nature of the training and illness data is potentially a major limitation. The use of self-reporting might also have resulted in mild illness symptoms or episodes being missed. While Sylta et al. [24] have previously demonstrated accurate self-reporting of endurance training among elite-level XC skiers, the athletes in their study were senior national-team members who were considerably older (20–32 y) than the skiers in the current study (16–22 y). It is possible that age and experience would have reduced the accuracy of reporting in the present study, which would have had a greater impact during the earlier parts of the investigated period. However, all athletes were followed-up closely by high school, university, and/or national-team coaches throughout the observation period, which should have reduced the likelihood of inaccurate reporting.

Secondly, it should be noted that the current study does not include data on forms of training other than endurance training, such as strength, power, and sprint training, due to a lack of consistent reporting routines. Previous investigations have demonstrated that training forms other than endurance training comprise a relatively small amount of the total training volume performed by elite XC skiers (~ 5%) [2], but that world-class skiers perform a larger amount of both strength and speed training compared to national-level skiers [6]. Hence, future investigations should explore the characteristics and importance of these training forms in the development of young XC skiers in relation to both distance and sprint XC skiing performance.

Thirdly, data relating to symptoms of illnesses and/or physician verification were not available in the present study. Hence, it was not possible to determine any causes of the self-reported illness episodes. While any reduction in or loss of training time as a consequence of illness is important, regardless of cause [10, 44], future studies should aim to determine the causes of reported illness episodes in developing XC skiers. This may enable the subsequent reduction of developing illness within this population.

## Conclusions

This study provides novel insights into the training and illness characteristics of young, well-trained XC skiers throughout a critical phase of their development. The skiers progressively increased their endurance training volume in a linear fashion from age 16 to 22 y. The increase in endurance training volume was primarily achieved through an increase in LIT and sport-specific training, rather than HIT and non-specific training. The developing skiers in the present study reported an average of 3 illness episodes per year, each typically lasting 4 days, which is comparable to senior elite-level athletes and the general population. In addition, higher training volumes were associated with a lower number of self-reported illness days, supporting the notion that high training volumes are compromised with more lost training days due to illness. Further studies investigating the optimal progression and structure of endurance training of young developing XC skiers would be beneficial, as would a systematic analysis of the likely causes of illness episodes. This study has also highlighted the need for better reporting routines of training forms other than endurance training, such as speed, strength, mobility/flexibility and illness episodes.

## Supporting information

S1 File Raw data. (XLSX)
